# Strongyloidiasis infection in a borderline lepromatous leprosy patient with adrenocorticoid insufficiency undergoing corticosteroid treatment: a case report

**DOI:** 10.1186/s13256-022-03673-4

**Published:** 2022-12-09

**Authors:** Rumala Morel, Kusala Maddumabandara, Nisansala Amarasinghe, Sujeewa Amarangani, Anjalie Amarasinghe, Mihiri Gunathilaka, Gayani Wathsala, Lakmalee Bandara, Sunil Wijesundara, Nilupuli Gunaratne, Roshitha Waduge, Arjuna Medagama

**Affiliations:** 1 Department of Parasitology, Faculty of Medicine, University of Peradeniya, Peradeniya, Kandy, Sri Lanka; 2 Department of Medicine, Faculty of Medicine, University of Peradeniya, Peradeniya, Kandy, Sri Lanka; 3 Department of Pathology, Faculty of Medicine, University of Peradeniya, Peradeniya, Kandy, Sri Lanka

**Keywords:** *Strongyloidis stercoralis*, Leprosy, Corticosteroid therapy, Adrenocortical insufficiency, Sri Lanka

## Abstract

**Background:**

Strongyloidiasis is a soil-transmitted helminthiasis mainly caused by *Strongyloides stercoralis*. It is endemic to the tropics and subtropics. Sri Lanka has a 0–1.6% prevalence rate. *S*. *stercoralis* infection was identified in a 33-year-old Sri Lankan male patient treated with corticosteroids for borderline lepromatous leprosy with adrenocortical dysfunction.

**Case presentation:**

In March 2020, a 33-year-old Sri Lankan (Sinhalese) male patient presented with watery diarrhea, lower abdominal pain, and post-prandial abdominal fullness. Previously, he was diagnosed with borderline lepromatous leprosy and was treated with rifampicin, clofazimine, and prednisolone 60 mg daily since July 2019. After developing gastrointestinal symptoms, he had defaulted leprosy treatment including the prednisolone for 3 months. Duodenal biopsy revealed numerous intraepithelial nematodes within the lumina of glands in the duodenum whose appearance favored *Strongyloides*. Fecal wet smear revealed numerous *Strongyloidis stercoralis* L1 rhabditiform larvae. Larval tracks were seen in the agar plate culture. L3 filariform larvae of *Strongyloidis stercoralis* were seen in the Harada–Mori culture.

In addition, the short synacthen test revealed adrenocortical insufficiency, and oral hydrocortisone and fludrocortisone were started with albendazole treatment against strongyloidiasis. Fecal wet smear and culture repeated after treatment with albendazole were negative for *Strongyloidis stercoralis*. The patient was discharged in July 2020 on oral hydrocortisone. One month later his condition was reviewed and the repeated fecal wet smear and agar plate culture was normal. He is being followed up every 3 months.

**Conclusion:**

This is the first case of strongyloidiasis diagnosed in a patient with borderline lepromatous leprosy from Sri Lanka. The patient manifested symptoms of strongyloidiasis while on high-dose steroid therapy for his lepromatous reaction. Subsequently, the patient not only discontinued his steroid therapy, but also developed adrenocortical insufficiency as a complication of leprosy. Therefore, although diagnosis of strongyloidiasis was delayed, his subsequent low steroid levels probably protected him from disseminated disease. This is an interesting case where symptomatic strongyloidiasis was diagnosed in a patient who was initially treated with high-dose steroids but subsequently developed adrenocortical insufficiency. We emphasize the need to screen all patients prior to the commencement of immunosuppressive therapy.

## Introduction

Strongyloidiasis is a soil-transmitted helminthiasis mainly caused by *Strongyloidis stercoralis* and, on rare occasions, by *Strongyloides fuelleborni fuelleborni* and *Strongyloidis fuelleborni kelleyi* [1]. Soil-dwelling filariform larvae can penetrate through the human skin when the host walks barefoot on contaminated soil. Filariform larvae migrate to the lungs, then into the small intestine and become adults. The female adult worms release eggs into the gastrointestinal tract [2, 3]. Strongyloidiasis is endemic to tropical and subtropical countries [1]. Sri Lanka which is a tropical country, has a 0–1.6% prevalence rate [4]. Previous cases report strongyloidiasis in transplant patients and immunocompromised patients in Sri Lanka [5–7]. Corticosteroids have a particularly strong and specific association with the development of hyperinfection syndrome and dissemination [8].

We report a case of strongyloidiasis in a patient with borderline lepromatous leprosy who was given corticosteroids to treat his lepromatous reaction. He later developed severe adrenocorticoid insufficiency, which necessitated placing him on lifelong steroids.  This is an interesting case where strongyloidiasis was diagnosed in a patient who was previously on high dose steroids but then developed adrenocorticoid insufficiency and therefore had a low steroid level. To our knowledge this is also the first case report of strongyloidiasis in a patient with leprosy in Sri Lanka.Together with a literature review, this case reports *S*. *stercoralis* infection that was identified in a 33-year-old male patient who was on corticosteroids while being given anti-leprosy drugs.

## Case presentation

In March 2020, a 33-year-old Sri Lankan (Sinhalese) male patient presented with watery diarrhea, lower abdominal pain, and post-prandial abdominal fullness to a rural hospital in the North Central Province of Sri Lanka. He was a member of the Civil Defense Force. Although the patient was transferred to a District General Hospital from the rural hospital, they were unable to diagnose the cause of his severe gastrointestinal symptoms. After 3 months he was transferred to a tertiary hospital in the Central Province, which was 170 km away. The patient had 3–4 episodes of loose stools per day, mainly of food particles without any blood or mucus. Previously, he was diagnosed with borderline lepromatous leprosy and was treated since July 2019 with rifampicin, clofazimine, and 60 mg prednisolone daily. While he was on those drugs, he developed severe gastrointestinal symptoms and therefore defaulted leprosy treatment for 3 months.

The patient had severe loss of appetite and lost 15 kg of body weight during these 3 months. He did not have vomiting or nausea but had lower abdominal pain with post-prandial abdominal fullness. He denied cough, fever, shortness of breath, night sweats, urinary symptoms, dysuria, and joint pains. On examination, the patient was cachectic and pale. His body weight was 32 kg and he had no skin rashes. There was mild left iliac fossa tenderness with no hepatosplenomegaly.

His white blood cell count was high (14.54 × 10^3^/µL) with neutrophil predominance (10.2 × 10^3^/µL). The eosinophil count was normal. Hemoglobin level was marginally low (10.20/ µL) and the platelet counts were normal (436 × 10^3^/µL). C-reactive protein was normal (2.8 mg/L), sodium (Na^+^) level was low (119 mmol/L), and potassium (K^+^) was in the lower margin of the normal range (4.20 mmol/L). Urinary Na^+^ (47 mEq/L) and urinary K^+^ (23.5 mEq/L) were high. His serum creatinine was normal (50.4 µmol/L). Total protein was low (38.4 g/L) with a very low albumin level (17.5 g/L). Aspartate aminotransferase test (AST), alanine aminotransferase test (ALT), and international normalized ratio (INR) were normal. The blood picture was compatible with anemia of chronic disease.

His Mantoux test was negative, and Hepatitis B and C, as well as human immunodeficiency viruses (HIV) tests were also negative. His CD4, CD8, and CD19 cell counts were normal. The short Synacthen test was positive (9.00 am—24 nmol/L; 9.30 am—12 nmol/L) and adrenocortical insufficiency was diagnosed. A short Synacthen test is used for diagnosis of adrenocortical insufficiency on the basis of the measurement of serum cortisol before and after injection of synthetic adrenocorticotropic hormone (ACTH). A cortisol level of more than 420 nmol/L at 30 min post-Synacthen indicates an adequate adrenal response. Computed tomography (CT) scan of the chest and abdomen was normal except for bilateral small adrenal glands, which did not show any calcifications.

The patient was started on oral steroids (oral hydrocortisone 35 mg daily and fludrocortisone 100 µg daily) in June 2020. Complement factors C3 was normal (71 mg/dL), C4 was marginally low (19 mg/dL), immunoglobulin G (IgG) was normal (966 mg/dL), immunoglobulin M (IgM) was low (39 mg/dL), and immunoglobulin A (IgA) was normal (209 mg/dL).

**Fig. 1:**
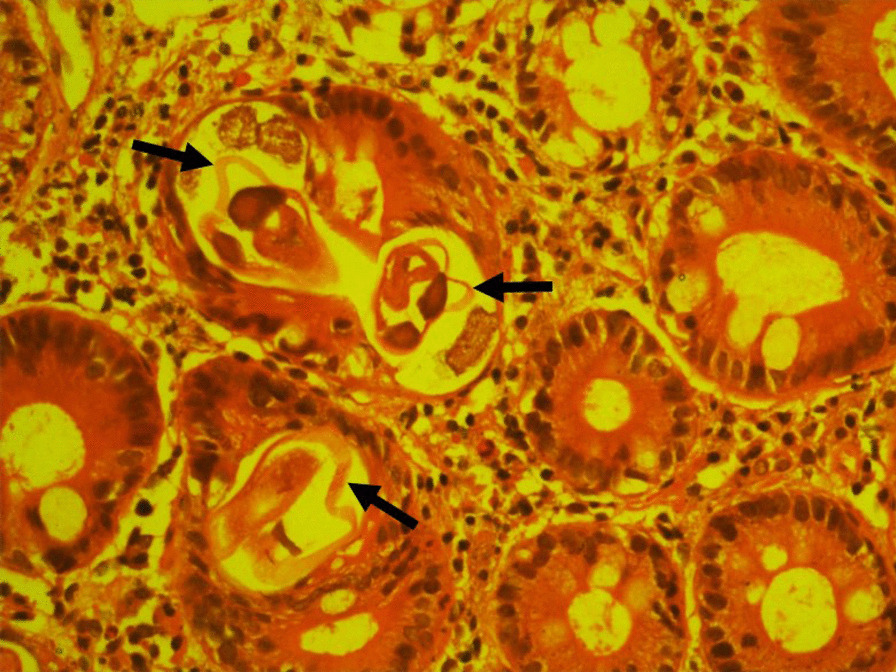
Intraepithelial nematodes are indicated with arrowheads observed in the duodenal (D2) biopsy within the lumina of glands in the duodenum. Duodenal biopsy—arrowheads indicate cross sections of intraepithelial nematodes (*S. stercoralis* larvae) within the lumina of glands in the duodenum

**Fig. 2:**
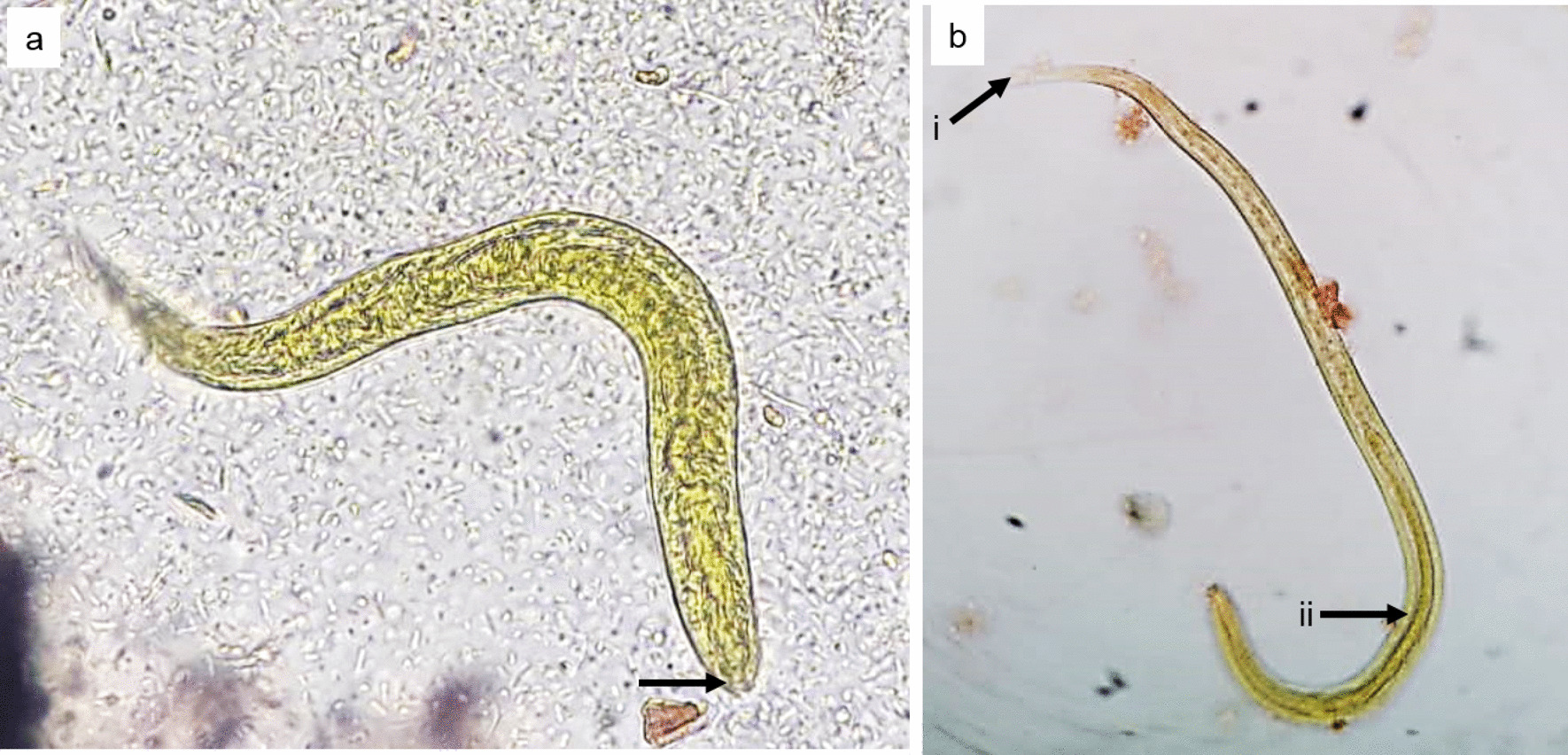
**a*** S. stercoralis* L1 rhabditiform larva seen in the fecal wet smear. The arrowhead indicates shallow buccal cavity.** b** L3 filariform larva of* S. stercoralis* seen in the Harada Mori culture. Arrowheads indicate (i) the notched tip of the tail and (ii) the oesophagus which is half of the body length of the larva

Owing to persisting diarrhea, upper gastrointestinal endoscopy (UGIE), duodenal biopsy, colonoscopy, and biopsy were performed. A severely inflamed oedematous duodenum was noted. The ileum was not inflamed and the gross appearance was normal. Duodenal (D2) biopsy revealed numerous intraepithelial nematodes within the lumina of glands in the duodenum, and the appearance was that of parasitic infestation favoring *Strongyloides* spp. (Fig. 1). Biopsies taken from the ileum, caecum, and the rest of the colon were normal. Fecal wet smear revealed numerous *S. stercoralis* L1 rhabditiform larvae (Fig. 2a). Coproculture using the Harada–Mori technique yielded L3 filariform larvae, which confirmed the species identification by its long esophagus and the tail with a notched tip (Fig. 2b). Larval tracks were seen in the agar plate culture of feces. The patient was diagnosed with strongyloidiasis and was treated with albendazole 400 mg twice a day for 7 days according to the CDC, USA treatment guidelines.

## Outcome and follow-up

With this treatment, his appetite improved and he was started on a high-protein diet. Oral hydrocortisone (daily dose 35 mg) and fludrocortisone 100 µg daily were continued. Fecal wet smear and agar plate culture repeated after treatment with albendazole were negative for *S. stercoralis*. The patient was discharged on 20 July 2020 on oral hydrocortisone (daily dose 25 mg). One month later, in August 2020, his condition was reviewed. The repeated fecal wet smear and culture did not reveal *S.stercoralis* larvae. Fecal smears and agar plate cultures carried out on the patient’s wife and child were also normal. Patient continues to be followed up every 3–6 months as he is on lifelong steroid therapy. He was also advised to use footwear.

## Discussion and conclusions

*Strongyloides stercoralis* is a unique intestinal helminth species with a self-perpetuating life cycle. This is responsible for its potential lifelong infection and capacity to kill its human host. It is estimated that 30–100 million people are infected worldwide [1]. Potentially life-threatening disseminated strongyloidiasis in immunocompromised patients results in mortality rates of up to 85% [8]. Strongyloidiasis is usually asymptomatic and often only indicated by a raised eosinophil count. However, it can present with dermatological or gastrointestinal symptoms [3].

Steroids administered to treat underlying conditions represent the main trigger predisposing to *Strongyloides* hyperinfection syndrome (SHS) and disseminated strongyloidiasis [9, 10]. SHS represents the increased number of larvae within the traditional reproductive route (skin, GI tract, lungs), while disseminated strongyloidiasis involves widespread dissemination of larvae outside the GI tract and lungs (liver, heart, brain, urinary tract) [10]. Increased number of larvae in stool and sputum is the hallmark of SHS [8]. The gastrointestinal manifestations include severe abdominal cramping, pain, watery diarrhea, nausea, vomiting, weight loss, occasionally gastrointestinal bleeding, small bowel obstruction, and pulmonary manifestations with cough, wheezing, pulmonary hemorrhage, pleural effusion, and acute respiratory failure [2]. Bacteria carried with the larvae into the bloodstream can lead to gram-negative sepsis which may be fatal, especially in immunosuppressed patients [11].

This patient is on 25 mg hydrocortisone daily. A high dose of steroids is defined as more than 15–20 mg for longer than 2–4 weeks [12]. However, dose, duration, or route of administration of drugs is not related to SHS, as even a short course of steroids (6–17 days) in immunocompetent patients can be associated with fatal SHS [8].

Previous cases reporting *Strongyloides* infection in patients with leprosy undergoing steroid treatment have been identified from Asia [13–15]. Patients with leprosy and *S. stecoralis* infection have developed SHS due to steroids being administered to patients with leprosy[16, 17]. The patient that we report in this case discontinued his leprosy treatment, including prednisolone, owing to his severe gastrointestinal symptoms. Thereafter, he developed adrenocortical insufficiency. The resulting low cortisol levels probably protected him from developing SHS. Thus, suffering a complication of leprosy protected this patient from complications due to infection with *S. stercoralis*. Subclinical adrenocortical insufficiency has been reported in patients with leprosy [18]. This patient, however, developed severe adrenocortical insufficiency with reduced volume of bilateral adrenal glands, which necessitated long-term steroid therapy.

*Strongyloides stercoralis* can cause chronic eosinophilia in humans owing to auto-infection within the human host [19]. Although this patient had increased neutrophils, his eosinophil count was normal. Corticosteroids can reduce the levels of circulating eosinophils by inhibiting their proliferation and increasing apoptosis [20].

Fecal examination for *Strongyloides* larvae and enzyme-linked immunosorbent assay (ELISA) for IgG antibodies, as well as eosinophilia in peripheral blood, are recommended to screen kidney donors and recipients. Since *S. stercoralis* can be dormant in an infected person and can cause SHS when the patient becomes immunocompromised, patients with strongyloidiasis should be treated before transplantation and commencement of immunosuppressive therapy [21, 22]. Therefore it is important to continue investigating for strongyloidiasis in this patient, as he is on lifelong treatment for adrenocortical insufficiency and longstanding chronic infection is well recorded [23–25].

Strongyloidiasis is mainly diagnosed by the detection of larvae found in feces. Coproculture techniques such as Harada–Mori filter paper, charcoal, and agar plate cultures are more labor intensive but have a higher sensitivity than direct fecal smears [26]. These techniques are used to specifically differentiate *S. stercoralis* infection from other intestinal nematode infections [27, 28]. In this case the diagnosis of strongyloidiasis was delayed for more than 3 months. Fecal examination for *S. stercoralis* was done only after the duodenal (D2) biopsy showed intraepithelial nematodes within the lumina of glands in the duodenum (Fig. 1).

This patient was treated with albendazole 400 mg twice a day for 7 days while continuing his steroid therapy. Several drugs have been recommended for the treatment of strongyloidiasis, such as albendazole, mebendazole, thiabendazole, and ivermectin. Ivermectin is more effective for SHS, primarily when associated with predisposing conditions such as acquired immunodeficiency syndrome [29]. A previous case [5] in Sri Lanka highlighted the importance of careful follow-up of cases after treatment. The patient in this case was reviewed after 1 month and the repeated fecal wet smear and culture were normal. We plan to continue to follow-up with him every 3 months, as he will be on lifelong steroid therapy owing to adrenocortical insufficiency.

This is the first case of strongyloidiasis diagnosed in a patient with borderline lepromatous leprosy from Sri Lanka. Strongyloidiasis was not considered in the differential diagnosis until the duodenal biopsy showed nematode larvae. If there is greater awareness of strongyloidiasis among clinicians, the patient could have been treated in the rural hospital where he was initially admitted. As it is, he had to suffer for 3 months until he was finally transferred to a hospital 170 km away.

This case also highlights an interesting situation where, in spite of the delay in diagnosis of strongyloidiasis, the patient was protected from disseminated disease owing to developing adrenocorticoid insufficiency as a complication of leprosy.

Although Sri Lanka is an endemic country for strongyloidiasis, the recorded prevalence is very low [4]. The Centers for Disease Control and Prevention (CDC), USA guidelines state that physicians should screen for strongyloidiasis prior to starting corticosteroids or other immunosuppressants [30]. This case emphasizes the need for such screening.

## Data Availability

The low prevalence rate of strongyloidaisis reported in Sri Lanka is due to poor diagnosis. Physicians do not suspect strongyloidiasis and therefore do not test for it. This is clearly shown in this case report where the patient suffered for six months and was transfered a long distance away to a tertiary hospital before he was diagnosed. If he was screened prior to starting immunosuppressive therapy this would not have happened.

## Abbreviations

LIF: Left iliac fossa
CRP: C-reactive protein
CDC: Centers for Disease Control and Prevention
Na^+^: Sodium ion
K^+^: Potassium ion
AST: Aspartate aminotransferase test
ALT: Alanine aminotransferase test
INR: International normalized ratio
HIV: Human immunodeficiency viruses
CECT: Computed tomography
IgG: Immunoglobulin G
IgM: Immunoglobulin M
IgA: Immunoglobulin A
UGIE: Upper gastrointestinal endoscopy
SHS: Strongyloides hyperinfection syndrome
ELISA: Enzyme-linked immunosorbent assay
